# An optimized methodology for whole genome sequencing of RNA respiratory viruses from nasopharyngeal aspirates

**DOI:** 10.1371/journal.pone.0199714

**Published:** 2018-06-25

**Authors:** Stephanie Goya, Laura E. Valinotto, Estefania Tittarelli, Gabriel L. Rojo, Mercedes S. Nabaes Jodar, Alexander L. Greninger, Jonathan J. Zaiat, Marcelo A. Marti, Alicia S. Mistchenko, Mariana Viegas

**Affiliations:** 1 Ricardo Gutiérrez Children’s Hospital, Ciudad Autónoma Buenos Aires, Argentina; 2 Consejo Nacional de Investigaciones Científicas y Tecnológicas (CONICET), Buenos Aires, Argentina; 3 Ministerio de Salud de la Ciudad de Buenos Aires, Buenos Aires, Argentina; 4 Department of Laboratory Medicine, University of Washington, Seattle, Washington, United States of America; 5 Argentine Bioinformatic Platform (BIA), Buenos Aires, Argentina; 6 Comisión de Investigaciones Científicas (CIC), Buenos Aires, Argentina; Kliniken der Stadt Köln gGmbH, GERMANY

## Abstract

Over the last decade, the number of viral genome sequences deposited in available databases has grown exponentially. However, sequencing methodology vary widely and many published works have relied on viral enrichment by viral culture or nucleic acid amplification with specific primers rather than through unbiased techniques such as metagenomics. The genome of RNA viruses is highly variable and these enrichment methodologies may be difficult to achieve or may bias the results. In order to obtain genomic sequences of human respiratory syncytial virus (HRSV) from positive nasopharyngeal aspirates diverse methodologies were evaluated and compared. A total of 29 nearly complete and complete viral genomes were obtained. The best performance was achieved with a DNase I treatment to the RNA directly extracted from the nasopharyngeal aspirate (NPA), sequence-independent single-primer amplification (SISPA) and library preparation performed with Nextera XT DNA Library Prep Kit with manual normalization. An average of 633,789 and 1,674,845 filtered reads per library were obtained with MiSeq and NextSeq 500 platforms, respectively. The higher output of NextSeq 500 was accompanied by the increasing of duplicated reads percentage generated during SISPA (from an average of 1.5% duplicated viral reads in MiSeq to an average of 74% in NextSeq 500). HRSV genome recovery was not affected by the presence or absence of duplicated reads but the computational demand during the analysis was increased. Considering that only samples with viral load ≥ E+06 copies/ml NPA were tested, no correlation between sample viral loads and number of total filtered reads was observed, nor with the mapped viral reads. The HRSV genomes showed a mean coverage of 98.46% with the best methodology. In addition, genomes of human metapneumovirus (HMPV), human rhinovirus (HRV) and human parainfluenza virus types 1–3 (HPIV1-3) were also obtained with the selected optimal methodology.

## Introduction

Complete genome sequences are a powerful tool for pathogen characterization, molecular surveillance, diagnosis, viral attenuation, response to drug treatment, response to host immune pressure and even new pathogen discovery [1–5]. In the last years, complete genome sequences published in databases have grown exponentially along with the development of more sensitive and faster sequencing technologies [6]. Currently, next generation sequencing (NGS) is one of the most reliable and most popular technologies for this purpose. One of the most interesting features of NGS is its ability to obtain detailed information about the pathogen in the sample just in one test in a relatively unbiased manner [7,8]. The strategy of NGS includes a wet lab phase in which the target virus can be enriched and a dry lab phase in which an accurate data analysis pipeline should be developed. The enrichment of the target virus is often carried out by viral culture or with PCR amplification by using specific primers [9–12]. Nevertheless, the genome of RNA viruses is highly variable and any kind of enrichment may be difficult to achieve or may bias the results selecting or enriching some viral variants over others. Currently, there are many publications which include methodologies to obtain complete viral genomes trying to avoid bias sources but there are not comparative evaluations among them[13–18]. The enrichment method used in some of these works was the RNA retrotranscription and cDNA amplification with random hexamers and in others with SISPA primers. In some cases, the clinical samples were filtered, treated with DNAse or subjected to a depletion of rRNA while in other cases combinations of these treatments were used. Each described treatment has been successful, but there is no in-depth analysis of which of them is really necessary. Furthermore, when working with clinical samples, the available volume is a major constraint. At the same time, it would be desirable that the number of samples to be sequenced be as large as possible, even more when molecular surveillance studies are carried out.

Within respiratory viruses, human respiratory syncytial virus (HRSV) is the main viral cause of acute lower respiratory tract infections in pediatric patients worldwide. The genome of HRSV is a negative sense ssRNA of approximately 15,200 nucleotides in length, which includes 10 genes encoding 11 proteins. Currently, several clinical trials with live-attenuated virus vaccines against HRSV have been reported and most of them are nearing completion, which makes promising the possibility of preventing the severe disease produced by this virus in a near future [19]. In this context, the global viral surveillance at the genome level is essential to verify whether the formulated strain will protect against global and local population of circulating viruses. This is important not only after vaccine implementation but also before to set a precedent of knowledge about the current ecology of the virus without any vaccine pressure.

In this work, we described an optimized methodology to obtain genomic sequences of HRSV (subgroups A and B) from clinical samples. We evaluated different sample treatments in order to enrich the viral RNA extracted from the respiratory clinical samples prior NGS library preparation. At the same time, we tested variations in the library preparation protocol to obtain the best performance in an economic manner. Furthermore, we described our bioinformatics workflow used for data analysis. Finally, we tested the optimal methodology to obtain nearly complete genome sequences of other RNA respiratory viruses (human metapneumovirus, human rhinovirus and human parainfluenza virus types 1–3).

## Materials and methods

### Ethics statement

The project was reviewed and approved by the Medical Ethics and Research Committees of Hospital de Niños “Ricardo Gutiérrez”, Buenos Aires, Argentina (IRB No. 17.21). Parental informed consent was not obtained because patient information was anonymized and deidentified before analysis.

### Clinical samples and RNA extraction

Clinical samples were nasopharyngeal aspirates (NPA) which resulted positive for HRSV by an indirect immunofluorescence assay as a standard care protocol for respiratory virus routine diagnosis [20]. Samples were taken from pediatric patients under 2 years of age hospitalized due to acute lower respiratory tract infection at the Ricardo Gutierrez Children’s Hospital (HNRG) in Buenos Aires, Argentina during the 2015 and 2016 outbreaks. The samples analyzed in this study were anonymized and were randomly selected from those that had viral load higher than E+06 viral copies/ml of NPA. The viral load detection was performed as previously described [21]. The NPAs were centrifuged at 5,000 g for 5 min to remove cellular debris. In certain cases, filtration through a 0.22 μm membrane filter was used after centrifugation to exclude the remaining cellular debris. Viral RNA was extracted from 200 μl of NPA with PureLink viral RNA/DNA mini kit (Thermo Fisher Scientific) following the manufacturer’s instructions except for 2.8 μl of carrier RNA instead of 5.6 μl as recommended to reduce the presence of tRNA in the extracted RNA. Viral-extracted RNA was eluted in 40 μl of DNase/RNase-free water and 1.25 μl of 40 U/μL RiboLock RNase Inhibitor (Thermo Fisher Scientific) were added to preserve the extracted RNA.

### DNase treatment

To remove the host DNA from samples, two different DNase treatments were performed: one was applied to the NPA before the RNA extraction and the other to the extracted RNA.

In the first treatment, 10 U of DNase I plus 20 μl of Buffer 10X with MgCl_2_ (Thermo Fisher Scientific) were added to 200 μl of NPA and were incubated at 37 °C for 1 hour. The reaction was stopped with 20 μl of 50 mM EDTA and was incubated at 65 °C for 10 min.

The second treatment was performed to 16 μl of the extracted RNA with the addition of 0.1 U of DNase I Amplification Grade (Thermo Fisher Scientific) and 2 μl of 10X Buffer. The reaction mix was incubated at room temperature for 15 min. This reaction was stopped with 2 μl of 25 mM EDTA and was incubated at 65 °C for 10 min.

### rRNA depletion

In certain samples, rRNA depletion to the extracted RNA was carried out to evaluate the decrease of host rRNA by using Ribo-Zero Gold rRNA Removal Kit (Epicentre, Illumina) according to manufacturer’s instructions.

### SISPA (Sequence-independent, single-primer amplification) [22]

Retrotranscription of RNA was performed with SuperScript III Reverse Transcriptase (SSIII) (Thermo Fisher Scientific). Briefly, 12 μl of viral RNA were mixed with 0.5 μl of 100 μM SISPA-A primer (5'-GTTTCCCAGTCACGATCNNNNNNNN-3') and 1 μl of 10 mM dNTPs. A denature step at 65 °C for 5 min was performed. Then, 4 μl of 5X Buffer, 1 μl of 0.1 mM DTT, 0.5 μl RNase Inhibitor (40 U/μl) and 200 U of enzyme were added (final volume 20 μl). Incubation conditions were 25 °C for 5 min; 50 °C for 60 min and 70 °C for 15 min.

The second strand cDNA was synthesized with DNA polymerase I, Large Fragment (Klenow) (New England Biolabs) by adding to the same tube of the retrotranscription reaction 1 μl of 10 mM dNTPs, 2 μl of 10X enzyme buffer, 1.5 μl of RNase/DNase-free H_2_O and 0.5 μl of enzyme (final volume of 25 μl). No additional primers were added. The incubation conditions were a denature step at 94 °C for 2 min before the enzyme addition and then 30 min at 37 °C followed by 20 min at 75 °C. Finally, the amplification of 5 μl of the double stranded cDNA (ds cDNA) by using 0.8 μM SISPA-B primer (5'-GTTTCCCAGTCACGATC-3') was performed with GoTaq DNA Polymerase (Promega) at a final volume of 25 μl following the manufacturer’s instructions. Amplification conditions were 95 °C for 5 min, followed by 35 cycles of 95 °C for 1 min, 65°C for 1 min and 72 °C for 2 min, and a final extension of 72 °C for 10 min. To increase the yield of ds cDNA, the reaction was performed in triplicate.

### Random hexamer priming without PCR amplification prior to library generation

The synthesis of the ds cDNA was performed as described in the previous section but random hexamers (Qiagen) at a final concentration of 1.6 μM were used instead of SISPA-A primers. In this case only the retrotranscription with SSIII and the ds cDNA synthesis with Klenow were performed.

### Purification and quantification of DNA

To purify the amplified ds cDNA, the DNA Clean & Concentrator-5 (Zymo Research, Epigenetics) was used following the manufacturer’s instructions. The ds cDNA was eluted with 12 μl of Tris-HCl pH 7.5–8.5 to maximize the performance of the elution. The DNA was quantified with Qubit 2.0 Fluorometer by using Qubit dsDNA HS Assay Kit (Thermo Fisher Scientific).

### Library preparation and NGS sequencing

Nextera XT DNA Library Preparation kit (Illumina) was used following manufacturer’s instructions. Half of the volume of the reagents in the DNA tagmentation and index incorporation were tested to optimize the yield of the kit. When necessary, a previous ds cDNA dilution with Tris-HCl pH 7.5–8.5 was performed to reach the required concentration of 0.2 ng/μl to start the Nextera XT protocol. The clean-up step was performed with 40 μl (0.8X) of Agencourt AMPure XP beads (Beckman Coulter) to maximize the recovery of fragments of 300 bp in size. Libraries were quantified by qPCR with NEBNext Library Quant Kit for Illumina (New England Biolabs) following manufacturer’s protocol. The normalization of the libraries was carried out manually by diluting each one to the concentration recommended by the manufacturer. Sequencing was performed in three independent runs, the first one on a MiSeq platform and the next two on a NextSeq 500. A total of 23 libraries were equimolarly pooled and were loaded in a 500 cycle MiSeq Reagent Kit v2 (Illumina) and a pair-end sequencing (2×250 bp) was performed. A total of 40 and 80 libraries were equimolarly pooled in two independent runs and were loaded in a 300 cycle mid-output (2x150 bp) NextSeq Reagent kits v2 (Illumina).

### Data analysis, genome mapping and consensus sequence obtaining

The quality of the fastq files was evaluated by FastQC tool [23]. To avoid reads with low quality scores, the PrinSeq tool was used to remove poor quality base calls by trimming 15 nt in the 5' end and 15 nt in the 3' end (trim_left 15 trim_right 15) and filtering with a minimum quality value of 30 (min_qual_mean 30) [24]. The mapping process was performed in two steps by using BWA software [25]. The first step was to select the most appropriate reference sequence. For that purpose, a concatenation of genome sequences of different HRSV genotypes previously published in GenBank was used for a screening mapping. Then, the sequence with the highest genome coverage was selected as the optimal reference to performed the final mapping [26]. Finally, duplicated reads were deleted and consensus sequence was obtained from the BAM file by using markdup module from SAMtools utilities [27]. Host rRNA contamination was determined by mapping the filtered reads to human cytoplasmic and mitochondrial rRNA databases (GenBank accession number NT_167214 and NC_012920, respectively). Host DNA contamination was evaluated with Kraken software (genome reference GRCh38.p7) [28].

### Sanger sequencing

In two samples (HRSV/A/001 and HRSV/B/002), Sanger sequencing was performed by amplifying the viral RNA into 15 overlapped fragments which covered the complete genome by RT-PCR as previously described [21]. Purified PCR products were labeled with the BigDye Terminator v3.1 sequencing kit (Applied Biosystems) and were sequenced in an ABI3500 genetic analyzer (Applied Biosystems). The SeqScape Software v2.7 (Applied Biosystems) was used to analyze, assemble and generate the consensus nucleotide sequences.

### Sample nomenclature, methodology and GenBank accession numbers

The sample nomenclature indicates the virus (HRSV: human respiratory syncytial virus; HMPV: human metapneumovirus; HPIV: human parainfluenza virus types 1–3; HRV: human rhinovirus), subtype (if available) and the number of the strain (laboratory internal number).

Consensus sequences for each sample are available on GenBank. Accession numbers are KY883566-KY883572, MG773266- MG773276 and MG839543- MG839547, MG881840.

**Fig 1:**
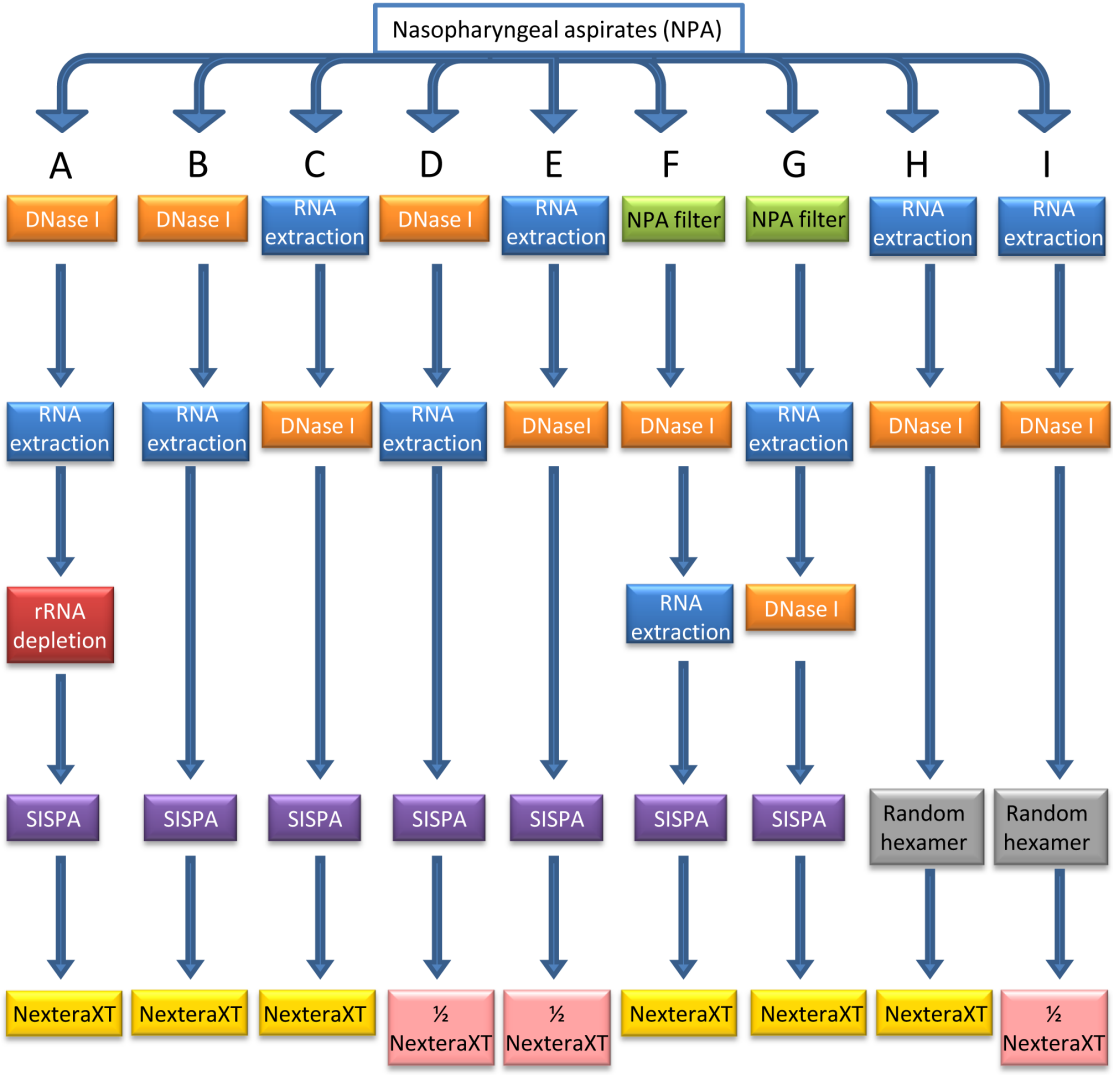
Scheme of sample treatments and library preparation workflow. Different treatments are combined in the methodologies which are denoted with the letters A to I.

Different combinations of treatments were named as A to I methodologies (Fig 1). Briefly, each one included: A- DNase treatment to the NPA, RNA extraction, rRNA depletion, SISPA and Nextera XT library preparation; B- A methodology without rRNA depletion; C- RNA extraction, DNase treatment to the RNA, SISPA and Nextera XT library preparation; D- B methodology with half of the volume of the Nextera XT library preparation; E- C methodology with half of the volume of the Nextera XT library preparation; F- B methodology with a first filtering-NPA step; G- C methodology with a first filtering-NPA step; H- C methodology with random hexamer amplification and I- H methodology with half of the volume of the Nextera XT library preparation.

## Results

### rRNA depletion (A and B methodologies)

To obtain viral genomic sequences from NPAs two methods (A and B) were evaluated (Fig 1). The first one included a Dnase I treatment directly to the NPA, a RiboZero treatment to the extracted RNA and SISPA (A methodology) and was applied to HRSV/A/001 and HRSV/B/002 clinical samples. At the same time, HRSV/B/002 sample was subjected to B methodology, which was different from A in which no rRNA depletion was performed. All libraries were prepared with Nextera XT kit and were sequenced on a MiSeq. It is appropriate to mention that although the DNA concentration of the samples before library preparation was below the recommended by manufacturer the Nextera XT protocol was followed.

The MiSeq run total yield was 5.9Gb with an error rate of 0.98% and a percentage of bases with quality score upper or equal to 30 (Q≥30) of 72.0%. An average of 1.5% of the quality filtered reads mapped against the HRSV reference sequence (Table 1). The genome coverage was almost complete in the three analyzed libraries even without rRNA depletion (99.7% in HRSV/A/001-Alibrary and 99.8% in both HRSV/B/002-A and B). Moreover, the depletion of the rRNA in HRSV/B/002 with the A methodology in comparison to the B one showed a 1.5% decrease of rRNA reads. Probably, no substantial difference was observed due to the relatively low amount of rRNA in the original samples.

**Table 1:** Comparison of sequencing results applying different methodologies.

Sample	copies/ml of NPA^(a)^	Methodology ID^(b)^	Total filtered reads ^(c)^	%mapped reads to viral reference^(d)^	%Duplicated reads	%genome coverage^(e)^	Average depth of coverage^(f)^	Min/Max depth of coverage	%Reads aligned to host^(g)^	%Reads aligned to rRNA^(h)^
MiSeq sequencing										
HRSV/A/001	1.51E+08	A	469,660	1.27	2.37	99.7	37.60	0/134	78	10.44
HRSV/B/002	4.13E+08	A	635,184	1.58	3.60	99.8	65.30	0/249	95	13.59
		B	796,524	1.50	3.30	99.8	84.70	0/297	96	15.64
NextSeq 500 sequencing										
HRSV/A/003	9.49E+06	B	1,378,092	5.62	91.21	96.5	49.20	0/227	58	20.11
HRSV/B/004	9.91E+07	B	2,317,914	4.90	93.18	98.6	56.03	0/230	25	24.00
		C	1,899,144	10.26	93.63	99.7	89.30	0/234	13	31.01
		D	3,054,972	4.86	94.81	96.9	55.60	0/233	19	22.22
		E	3,967,890	6.81	96.56	97.2	64.60	0/234	11	30.74
HRSV/B/005	9.38E+07	F	2,209,306	6.19	95.02	91	49.80	0/237	11	23.08
		G	2,121,050	21.66	97.29	99.8	90.50	0/239	18	24.64
HRSV/B/006	2.38E+08	C	904,954	80.62	98.11	99.7	97.70	0/239	63	9.78
		E	4,571,192	79.42	99.51	99.6	124.20	0/240	61	8.87
		H	460,408	1.24	34.85	99.4	22.30	0/67	97	9.21
		I	2,391,816	1.02	65.84	99.8	50.40	0/119	95	9.59
HRSV/A/007	2.40E+08	C	200,216	27.33	86.31	96.2	52.14	0/205	83	12.54
		E	353,266	30.28	91.75	94.36	59.05	0/223	80	12.77
		H	1,315,052	0.03	7.08	53.33	1.74	0/13	99	6.86
		I	1,914,840	0.03	13.51	51.72	2.19	0/35	98	14.04
HRSV/B/008	2.32E+08	C	157,926	11.77	80.18	94.04	25.33	0/192	93	13.44
		E	216,860	12.22	86.10	88.75	23.57	0/189	91	13.26
		H	1,513,366	0.08	14.32	93.61	6.19	0/25	98	11.99
		I	4,150,692	0.07	30.27	93.96	10.99	0/52	97	12.62
HRSV/A/009	4.53E+07	C	1,170,558	4.91	97.92	99.91	83.73	0/237	75	9.72
		E	617,392	41.37	96.44	97.14	61.29	0/237	74	8.95
		H	715,448	0.06	5.32	56.69	2.07	0/17	98	11.08
		I	1,563,020	0.05	11.93	79.85	3.58	0/22	97	11.43
HRSV/A/010	3.51E+08	C	357,194	50.62	89.96	99.76	122.23	0/210	50	14.77
HRSV/B/011	1.28E+08	C	478,062	62.88	95.64	99.93	93.60	0/239	67	17.42
HMPV/001	1.31E+08	C	1,448,584	1.88	81.82	99.03	36.93	0/179	84	24.22
HMPV/002	3.94E+07	C	121,464	0.75	15.27	86.82	5.11	0/41	49	9.56
HPIV1/001	6.14E+07	C	981,338	0.99	77.13	62.86	15.02	0/139	93	80.00
HPIV1/002	2.84E+09	C	1,066,666	68.06	97.29	99.9	143.09	0/236	49	15.20
HPIV2/001	5.51E+10	C	1,838,012	82.36	98.41	100	178.92	2/230	47	14.00
HPIV2/002	1.43E+08	C	518,934	18.02	90.01	96.67	64.79	0/181	36	10.34
HPIV3/001	1.97E+08	C	528,358	35.63	93.92	98.12	81.60	0/211	48	10.09
HPIV3/002	9.34E+08	C	9,293,908	95.37	99.70	99.98	204.30	0/241	44	0.30
HRV/C/001	9.59E+07	C	1,146,828	19.67	95.62	98.21	123.76	0/185	28	9.09

^(a)^ NPA: nasopharyngeal aspirate.

^(b)^ Description of A-H methodologies are in the manuscript and Fig 1.

^(c)^ Reads were filtered according quality scores upper or equal than 30.

^(d)^ For HRSV, GenBank accession number of the reference sequences are KU950583 for subtype A and JX576745 for subtype B; for HMPV is KF530179; for HRV is GU219984; for HPIV are KF530212, MF077313 and KJ672618 for types 1, 2 or 3, respectively.

^(e)^ Percentage regarding the length of each the reference sequence.

^(f)^ Average depth of coverage was calculated without duplicated reads.

^(g)^ Human reference: GRCh38.p7.

^(h)^ Human cytoplasmic and mitochondrial rRNA: NT_167214 and NC_012920.

**Fig 2:**
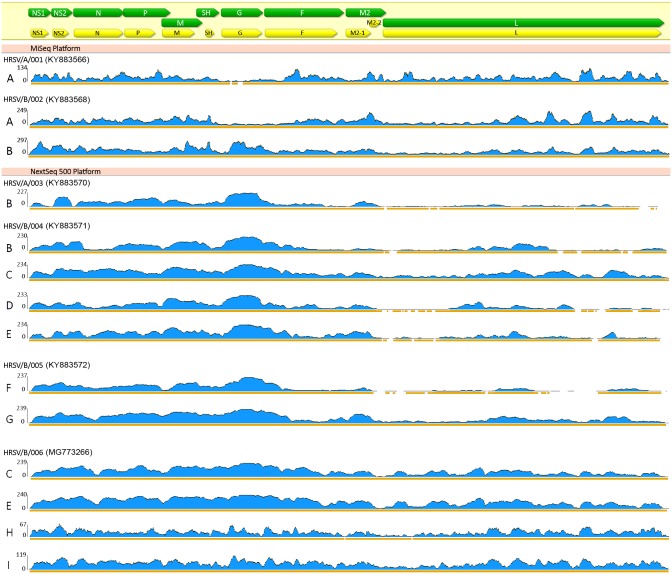
Coverage profiles of HRSV obtained per sample per methodology. A-I methodologies are indicated at the left of each profile. Genome organization with genes in green and their coding region in yellow are shown at the top of the figure. Genome regions with depth of coverage upper than 4 are underlined in orange. GenBank accession numbers are indicated in brackets. Only 6 representative samples are shown, other profiles are shown in S1 Fig.

The average depth of coverage was 37.6X, 65.3X and 84.7X for HRSV/A/001-A, HRSV/B/002-A and HRSV/B/002-B, respectively. The A methodology showed a profile of genome depth of coverage (from now on "coverage profile") with a minimum in the G gene sequence, which is the highest variable gene of the genome. In contrast, B methodology showed the highest depth of coverage in the G gene, reflecting a possible bias generated during the rRNA depletion procedure (Fig 2).

The analysis of host DNA percentage revealed that more than 75% of the reads of each library mapped to human DNA despite the DNase I treatment (78% in case of HRSV/A/001; 95% and 96% in case of HRSV/B/002-A and HRSV/B/002-B, respectively).

To evaluate the reliability of the methodology, we sequenced the samples HRSV/A/001 and HRSV/B/002 by Sanger. The nucleotide sequences for each sample were identical by both Sanger and NGS sequencing (GenBank accession number: KY883567 and KY883569), but NGS sequencing with SISPA covered more nucleotide bases from 5' and 3' end of the genomes.

### An improvement of RSV genome sequencing approach (C to I methodologies)

Different treatment combinations were evaluated to improve the sensitivity and specificity in HRSV sequencing reducing the percentage of human genome reads, improving the coverage profile across the genome and to increasing the cost-effectiveness as much as possible (Fig 1). Based on previous results, no rRNA depletion was considered for the new procedures. In this opportunity, all the libraries were sequenced on a NextSeq 500 platform in two independent runs. In the first run, the total yield was 38.6Gb with an error rate of 0.63% and 87.0% of the reads with Q≥30. The total yield in the second run was 59.3 Gb with an error rate of 2.29% and 68.7% of the reads with Q≥30.

#### Evaluation of DNase I treatment

To reduce the percentage of human genome reads, extracted RNA instead of NPA was treated with DNase I (C methodology). HRSV/B/004 was subjected to both B and C methods. The host contamination analysis showed that DNase I treatment to the extracted RNA was remarkably more efficient than to the NPA (Table 1). The reduction of host reads (from 25% to 13%) allowed to increase specific HRSV reads more than twice as the B methodology (from 4.90% to 10.26%).

At this point, it is important to mention that the use of a NextSeq 500 platform increased the capability to generate data; but in our experience this increment was related to the generation of many duplicated reads and most of them belonged to the G gene. For example, the HRSV/B/004-B library had an average depth of coverage for the G gene of 191.1X (minimum: 109X and maximum: 230X) but before removal of duplicated reads from the alignment it had an average depth of coverage of 9464.8X with a minimum of 448X and a maximum of 23263X (more information of the samples in S1 Table).

#### Evaluation of half amount of library preparation reagents

To increase the yield of library preparation reagents, the reactions of cDNA tagmentation and index incorporation were scaled down to half of the volume recommended by the manufacturer’s protocol [29,30]. The clean-up was performed according to the Agencourt AMPure XP beads protocol and normalization of libraries was performed manually, as was done with all methodologies. This reduction of reagents was tested either with DNase I treatment to the NPA or to the RNA (identified as D and E methodologies, respectively). The average depth of coverage was similar to those obtained with standard reaction volumes (55.6X, min/max: 0/233 for HRSV/B/004-D and 64.6X, min/max:0/234 for HRSV/B/004-E) and the coverage profiles were also similar. Thus, the reduction of reagents is possible and it allows increasing the number of libraries that can be prepared with one Nextera XT Kit (Table 1 and Fig 2).

#### Evaluation of 0.22 μm filtering

To test another approach to avoid the high presence of host DNA, the NPA was filtered with 0.22 μm pore-size membrane. This methodology was tested combined either with DNase I treatment to the NPA or to RNA (identified as F and G, respectively). Both methodologies did not show differences in the percentage of host DNA (11% vs 18%, respectively); nevertheless, the G methodology increased the recovery of HRSV reads from 6.19% to 21.66%, which allow the assembly of a complete genome (Table 1). The main problem with the use of the filter treatment was the loss of volume of the sample during the procedure, this can be limiting with samples of small volumes such as pediatrics. Thus, without the use of the filter step, the sequencing of genomes directly from NPA will be carried out properly if a DNase treatment to the RNA is performed (C methodology).

#### Evaluation of random hexamers instead of SISPA primers

We suspected that SISPA could bias the coverage profile achieved with the different methodologies (A to G), with a minimum depth coverage at the beginning of the L gene and a maximum in the G gene (whether or not the duplicated reads were taken into consideration). Thus, random hexamer amplification was evaluated with the use of either standard and half volume reaction of Nextera XT library preparation reagents (identified as H and I methodologies, respectively). Four samples were tested (HRSV/B/006, HRSV/A/007, HRSV/A/008, HRSV/B/009) and the results are listed in Table 1. Only two samples showed good sequencing performance with around 93% and 99% of the HRSV genome covered (HRSV/B/008 and HRSV/A/006, respectively). The other two samples yielded among 51.82% and 79.85% of the genome covered with low average depth of coverage. The coverage profiles were different with the random hexamer amplification; they were more uniformly distributed across the genome than with SISPA. Nevertheless, the beginning of the L gene remained with low depth of coverage (Fig 2).

The consensus sequences obtained with SISPA and with random hexamer amplification were identical for each sample. Nevertheless, PCR step in SISPA is an important step to increase the HRSV copy number.

#### Assay of the optimal methodology with other RNA viruses

From all the methodologies, the C one was the optimum (Fig 1). Thus, it was used for sequencing other respiratory RNA viruses present in NPA positive samples for human metapneumovirus (HMPV), human rhinovirus C (HRV-C) and human parainfluenza virus types 1, 2 and 3 (HPIV1-3). Complete and nearly complete genome sequences were obtained. The viral loads of the different respiratory viruses in the clinical samples were upper than E+07 viral copies/ml. Detailed information of sequencing performance is shown in Table 1.

## Discussion

The methodology described in this work proved to be robust and accurate. Complete and nearly complete viral genomes of respiratory RNA viruses were obtained from NPA samples with both platforms MiSeq and NextSeq 500. No correlation between sample viral loads and the number of total filtered reads or mapped viral reads was observed.

A limitation of this work is that the small sample volumes obtained from pediatric patients did not allow subjecting one NPA sample to all the proposed methodologies. In addition, no other types of clinical samples were evaluated.

Most sequenced HRSV genomes allowed to obtain complete information of all genes, however some of them could not completely cover the 5 ‘and 3’ ends of the genome, which led the percentage of genome coverage shown in Table 1 does not reach 100% (e.g.: HRSV/B/006-C in "% genome coverage" in Table 1 and coverage profile in Fig 2). Similarly, this occurred with the other analyzed RNA viruses.

The optimal methodology to obtain genomic sequences of RNA viruses by NGS determined in this work was a DNase I treatment to the RNA directly extracted from the NPA, the retrotranscription and the amplification performed with SISPA primers and library preparation performed with Nextera XT DNA Library Prep Kit with manual normalization. Nevertheless, half amount of library preparation reagents may be used instead of that recommended by manufacturer.

No rRNA depletion was used as part of the entire protocol due to the low performance accomplished and taking into account that it is an expensive procedure. In addition, the obtained coverage profile changed considerably related to all the others, reducing significantly the coverage of the G gene, one of the main genes used for HRSV genotyping.

Regarding the amplification with random hexamers, stochastic results were obtained independently of the viral load of the samples, thus it was defined to continue using SISPA primers, at least for genomic studies.

Many works on viral metagenomics already exist but no specific one focuses in the comparison of different combinations of treatments for the use in NPA samples to obtain RNA viral genomes [3,16,26,31,32]. Here, an improvement of the sensitivity and specificity in HRSV sequencing was evaluated to reduce the percentage of human genome reads, to improve the coverage profile across the genome and to reduce the cost as much as possible.

Strikingly, as mentioned in the result section, with the use of a NextSeq 500 platform a higher proportion of duplicated reads was obtained. These duplicated reads could be generated during the sequencing process or during the final step of SISPA, which was used to increase the viral copy number and could be revealed with the high output of this platform. In addition, the duplicated reads increased substantially the computational demand of the genome analysis when they were considered. From our results, the overall percentage of the HRSV genome recovery was not affected by the presence or absence of duplicated reads, therefore, they should be eliminated for the analysis and the high performance of the NextSeq could be used to pool more genomes per run instead of increasing the depth of coverage of each sample.

Noteworthy SISPA generated a coverage profile with a maximum at the G gene and a minimum at the beginning of the L gene with blank regions in few samples. It has been proposed that this coverage profile may be an artifact of the specific tag connected at the 5' end of the N-octamer present in the SISPA-A primer and later used as SISPA-B primer. This tag could bias the annealing across the genome favoring the over-amplification of some regions. As was done by Rosseel et al, stretches of nucleotides of the tag across our genome sequences were mapped looking for possible annealing and no correlation with over-represented depth of coverage was found. Even thought, other described sources of bias due to the use of SISPA, as the extension of the N-oligomer, might have an effect [33]. Several recent studies showed high G gene copy numbers or highly abundant G mRNA both in vitro and in clinical samples [34–36], providing a potential source of bias during genome amplification.

A previous work reported that SISPA has the limitation that only works well with a minimum number of E+06 viral particles per ml of sample, otherwise a sample with smaller amount of viral particles would cause a large number of non-specific amplified sequences [37]. An alternative strategy could be the sequencing of overlapped amplicons that anneal in highly conserved regions and cover the full HRSV genomes [30]. We are currently working on this strategy for samples with viral loads under E+06 viral copies /ml of NPA [38]. Nevertheless, we believe that the use of specific primers designed from pre-existing sequence data might not detect emergent strains. Thus, we consider of high importance that each region of the genome should be covered at least twice by different amplicons as was done by Agoti et al [39].

Complete genome sequences of human metapneumovirus, human parainfluenza types 1–3 and human rhinovirus could be obtained, which support the idea that this methodology is reproducible in other RNA viruses present in NPA samples.

Finally, assays to harmonize the coverage profile remain pending. Nevertheless, the use of SISPA may decrease the possibility of specific primer bias and provides the opportunity of detecting any other known/unknown RNA virus at the same time.

## Conclusion

The development of this methodology has great implications for molecular epidemiology because the enrichment of the viral nucleic acid does not require specific amplification or a previous step of viral culture, which accelerates the process of genomic characterization of any new HRSV strain that would emerge. In addition, this methodology demonstrated to be versatile for other RNA viruses from nasopharyngeal aspirates.

The data presented in this work show that a metagenomic approach combined with NGS is useful for whole genome sequencing of respiratory viruses directly from NPA and would allow virology laboratories to carry out molecular surveillance programs at the genomic level in real time.

## Supporting information

S1 Fig Additional coverage profiles obtained per sample per methodology. C, E, H and I methodologies are indicated at the left of each profile and described in the manuscript. Sequenced virus and genome length are denoted in pink at the top of the coverages. Genome regions with depth of coverage upper than 4 are underlined in orange. GenBank accession number are indicated at the right when appropriate. HRSV: human respiratory syncytial virus, HMPV: human metapneumovirus, HPIV: human parainfluenza virus (types 1, 2 and 3), HRV: human rhinovirus.
(JPG)

S1 Table Minimum (Min), maximum (Max) and average (Avg) depth of coverage with and without duplicated reads per gene per sample sequenced with C methodology. (XLSX)
