# Supplementary figures and images for: An optimized methodology for whole genome sequencing of RNA respiratory viruses from nasopharyngeal aspirates
Source: PLoS One. 2018 Jun 25;13(6):e0199714. doi: 10.1371/journal.pone.0199714 (PMC6016902; doi:10.1371/journal.pone.0199714)

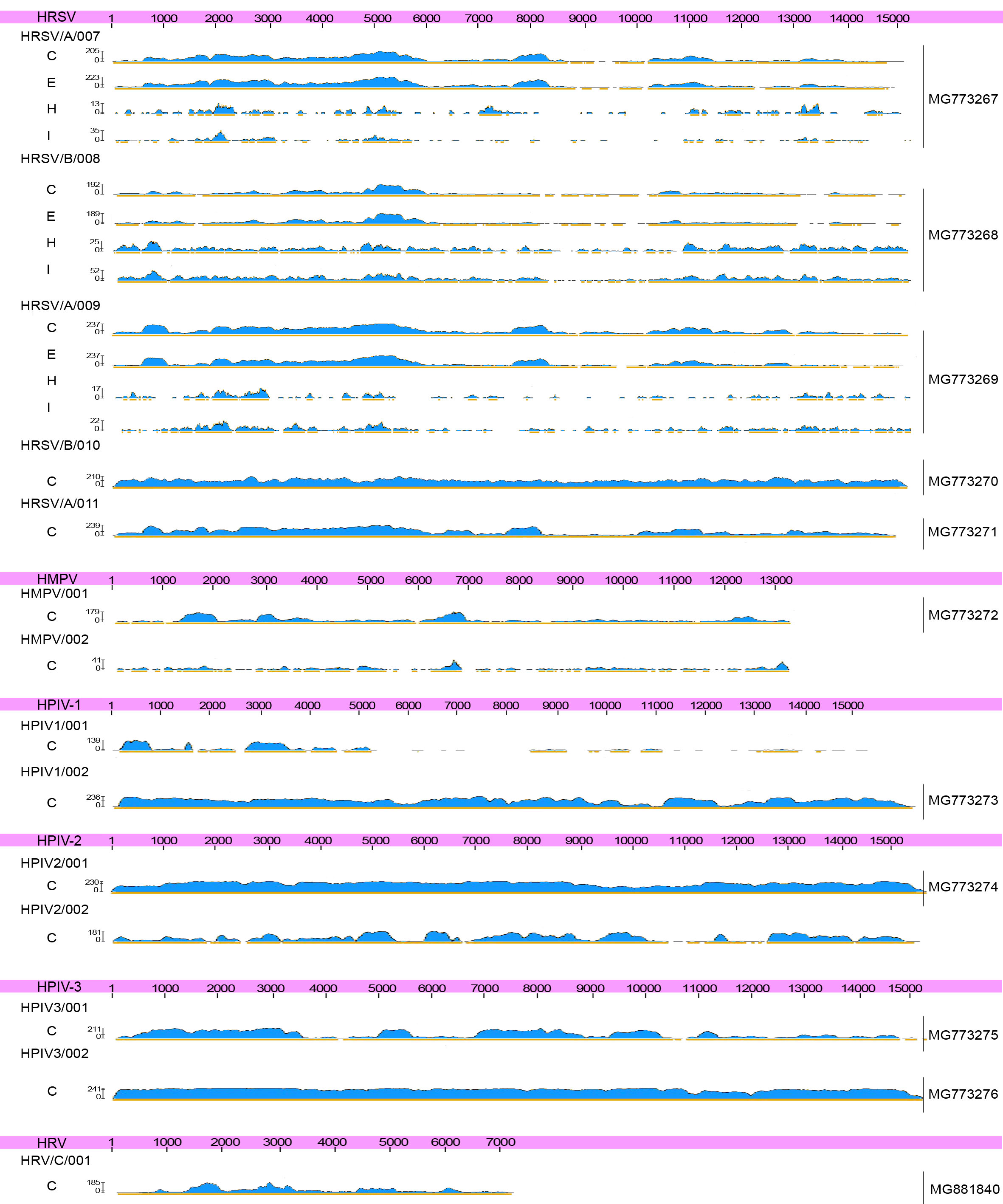

Supplement: S1 Fig — C, E, H and I methodologies are indicated at the left of each profile and described in the manuscript. Sequenced virus and genome length are denoted in pink at the top of the coverages. Genome regions with depth of coverage upper than 4 are underlined in orange. GenBank accession number are indicated at the right when appropriate. HRSV: human respiratory syncytial virus, HMPV: human metapneumovirus, HPIV: human parainfluenza virus (types 1, 2 and 3), HRV: human rhinovirus. (JPG) [file pone.0199714.s001.jpg]
